# Strategies for Targeted Delivery to the Peripheral Nerve

**DOI:** 10.3389/fnins.2018.00887

**Published:** 2018-11-27

**Authors:** Kelly A. Langert, Eric M. Brey

**Affiliations:** ^1^Department of Veterans Affairs, Research Service, Edward Hines, Jr. VA Hospital, Hines, IL, United States; ^2^Department of Biomedical Engineering, Illinois Institute of Technology, Chicago, IL, United States; ^3^Audie L. Murphy VA Hospital, San Antonio, TX, United States; ^4^Department of Biomedical Engineering, University of Texas at San Antonio, San Antonio, TX, United States

**Keywords:** drug delivery & targeting, peripheral nerve, nerve graft, blood-nerve barrier, nerve injury, inflammatory neuropathy

## Abstract

Delivery of compounds to the peripheral nervous system has the potential to be used as a treatment for a broad range of conditions and applications, including neuropathic pain, regional anesthesia, traumatic nerve injury, and inherited and inflammatory neuropathies. However, efficient delivery of therapeutic doses can be difficult to achieve due to peripheral neuroanatomy and the restrictiveness of the blood-nerve barrier. Depending on the underlying integrity of the blood-nerve barrier in the application at hand, several strategies can be employed to navigate the peripheral nerve architecture and facilitate targeted delivery to the peripheral nerve. This review describes different applications where targeted delivery to the peripheral nervous system is desired, the challenges that the blood-nerve barrier poses in each application, and bioengineering strategies that can facilitate delivery in each application.

## Introduction

**FIGURE 1:**
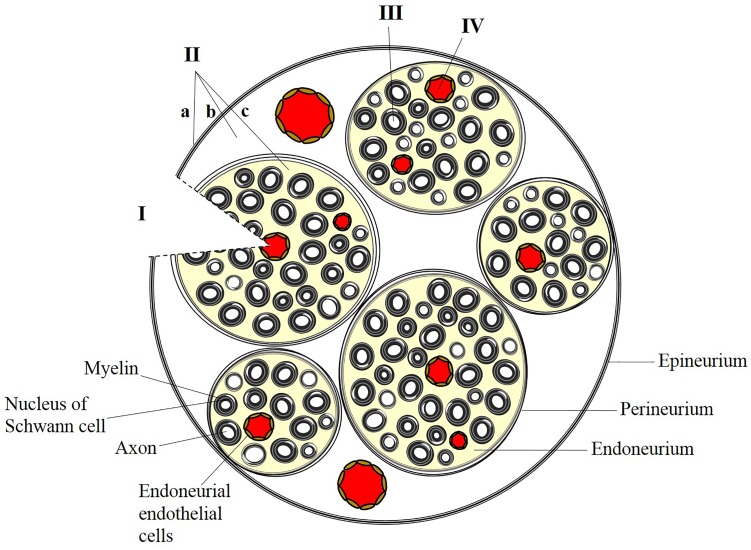
Strategies for targeted delivery to the peripheral nervous system. (I) Direct placement of a bioengineered nerve conduit in the presence of breached perineurial and blood-nerve (endoneurial) barriers. (II) Administration of anesthetic or analgesic by injection at or outside the epineurium (a), outside the perineurium (b), or within a fascicle (c). Administration of a peptide or protein requires localized, transient disruption of perineurial and blood-nerve barriers. (III) Presynaptic uptake and retrograde trafficking at peripheral nerve terminals results in delivery of cargo along individual axons within a fascicle. (IV) Endothelial targeting of systemically administered nanoparticles can preferentially target the endoneurial vasculature.

Delivery of therapeutic compounds to the peripheral nervous system (PNS) is difficult to achieve due to complexities of the peripheral neuroanatomy and the restrictiveness of the blood-nerve barrier (BNB). The endothelial cells that line the endoneurial vasculature are non-fenestrated and linked by specialized tight junctions, forming a restrictive barrier that protects the endoneurial microenvironment (Kanda, 2013) and ensures that molecules and ions in the systemic circulation will not interfere with sensory and motor signal transduction. Groups of axons and endoneurial blood vessels together form fascicles, which are further protected from the periphery by the perineurium, a component of the BNB composed of concentric layers of basement membrane and sheets of perineurial cells (Figure 1). The entire nerve is surrounded by the collagenous epineurium, which provides tensile strength but does not contribute to the BNB (Peltonen et al., 2013).

While the BNB is fundamental for normal function it hinders the transport of therapeutic compounds from the vasculature into the nerve. The sciatic nerve itself is impermeable to hydrophilic small molecules (Rechthand et al., 1987; Abram et al., 2006) as well as larger biologics (Liu et al., 2018); only small, lipophilic molecules can easily access the endoneurium. While this restrictiveness facilitates normal function, it is an issue in the clinic when considering delivery of therapeutics to peripheral nerves. The BNB serves as a major hurdle for access to drug targets that are in the peripheral nerves.

There are several scenarios where targeted delivery to the peripheral nerves is desired, and the features of BNB dictate the choice of an appropriate drug delivery strategy (Figure 1). For example, injury or traumatic damage to the nerve can breach the BNB. In these cases, regrowth of axons and repair of connections can be facilitated by local delivery of factors that promote a growth permissive environment (Madduri and Gander, 2012). Here, biomaterials can be directly implanted into the peripheral nerve environment providing bypassing the BNB. The biomaterials are a substrate for guided nerve regeneration while providing a platform for delivery of therapeutic molecules. These polymers can increase the half-life of labile growth factors and facilitate release at the site of injury, exploiting the loss of barrier integrity.

Local, controlled delivery at the site of repair also allows for administration of an experimental therapy that may otherwise be contraindicated as a systemic treatment (Lyons et al., 1994; Tajdaran et al., 2015; Langert et al., 2017; Nishihara et al., 2018). Mechanistic and preclinical studies frequently reveal novel therapeutic targets of interest; however, follow-up experiments often require high-dose, systemic administration (up to 100 mg/kg) of compounds that cannot be directly translated to the clinic (Han et al., 2014; Xiao et al., 2014; Pottabathini et al., 2016). In this regard, targeted delivery of therapeutic molecules to the PNS has the potential to increase therapeutic options for patients and minimize side effects.

In another example, local, controlled delivery of anesthetics or analgesics to peripheral nerves is necessary to provide a nerve block or relieve neuropathic pain. Here, the perineural and endoneurial barriers are intact and restrictive. Local anesthesia bears several advantages over general anesthesia for surgical procedures of the limbs (including the surgical repair of the injured nerves described above), and local peripheral nerve blocks can also be used to treat chronic neuropathic pain (Shankarappa et al., 2012). Strategies are necessary to facilitate diffusion of a compound across the intact perineurial and endoneurial barriers. In the case of conventional local anesthetics, strategies are necessary to minimize toxicity while providing an adequate nerve block.

Strategies based in gene therapy can also be exploited to target peripheral nerves in the presence of an intact barrier. Here, targeting can be conveyed by incorporating bacterial toxins that display natural neurotropism for ganglioside receptors that are enriched at presynaptic terminals. Retrograde transport can facilitate delivery of encapsulated cargo along the axon, to the dorsal root ganglia, or spinal cord. This approach has been investigated in models of regeneration (Lopes et al., 2017), inherited neuropathies (Wolfe et al., 2012), and neuropathic pain (Lopes et al., 2016).

In each of the aforementioned scenarios, the BNB poses a challenge in the delivery of therapeutic cargo (e.g., growth factors, small molecules, nucleic acids) to the endoneurial compartment. In this review, we describe bioengineering strategies that can be used for therapeutic, targeted delivery to the peripheral nerve. These strategies (illustrated in Figure 1 and outlined in Table 1) vary depending on the underlying integrity of the BNB, the properties of the agents to be delivered, and the nature of the associated clinical scenarios. We discuss advancements that have been made in preclinical studies and limitations in translating these findings to the clinic. Future opportunities for refinement of delivery strategies and the role of bioengineering in these opportunities are a matter of final discussion.

**Table 1:** Outline of strategies to access the endoneurium.

Strategy	I	II	III	IV
Status of BNB	Barrier breached	Barrier intact	Barrier intact	Barrier intact
Clinical example	Peripheral nerve trauma	Anesthesia, neuropathic pain	Gene therapy	Inflamed vasculature
Mechanism of access	Direct placement	Diffusion across BNB, with or without disruption	Retrograde axonal transport	Transendothelial migration


*The underlying status of the blood nerve barrier (BNB) and clinical application dictate the mechanism of access to the endoneurium.*

## Strategy I- Direct Placement at a Breached Barrier

The restrictiveness of the BNB is not a primary matter of concern when considering delivery after injury or trauma, as these events are associated with subsequent disruption of the BNB (Abram et al., 2006; Moreau et al., 2016). In addition, many injuries require surgical exposure of the nerve for assessment and repair. This provides an opportunity for direct placement of a graft or guide to facilitate healing and deliver proteins that would otherwise not be able to cross the BNB. Thus, access to the endoneurium and localized administration are not difficult to achieve in this scenario. Similarly, *in vivo* testing of drug delivery systems involves surgical exposure of the nerve at the time of injury modeling (typically crush or transection) allows for direct application of the nerve conduit or graft. Both clinically and experimentally, biomaterials incorporated into nerve conduits facilitate controlled release and protection of protein cargo, while providing structural support to regenerating axons.

Bioengineered nerve conduits can be synthesized from a broad range of natural [e.g., collagen (Catrina et al., 2013; Liodaki et al., 2013), laminin (Hsu et al., 2013), chitosan (Huang et al., 2011), alginate (Quigley et al., 2013)] and synthetic [e.g., poly (lactic-co-glycolic) acid (PLGA) (de Boer et al., 2012), polycaprolactone (PCL) (Reid et al., 2013)] biomaterials. Bioengineered grafts overcome concerns of available donor tissue and morbidity at the donor surgical site which is an improvement of the current standard of care, autologous nerve grafts, even in the absence of encapsulated factors. However, successful functional outcomes may be more frequently achieved with simultaneous delivery of trophic support in the form of growth factors and other growth-permissive pharmacological compounds (Piotrowicz and Shoichet, 2006; Madduri and Gander, 2012). Candidates for local delivery within a nerve conduit include nerve growth factor (NGF) (Lee et al., 2003), brain-derived neurotrophic factor (BDNF) (Zhao et al., 2014) and glial-derived neurotrophic factor (GDNF) (Fu et al., 2011).

While the basic structure of a bioengineered nerve conduit is a hollow tube, several strategies can be employed to incorporate cargo resulting in tunable release kinetics (Madduri and Gander, 2012). Different fabrication techniques can yield a tube filled with channels, longitudinal fibers (Dinis et al., 2015), or a matrix (Black et al., 2015). The lumen of the tube can be filled with growth factor-containing solution, a gel containing free growth factors or a gel containing polymeric microspheres providing a sustained delivery of factors. Alternatively, the inner wall of the nerve conduit can be coated with growth factor by physical adsorption, covalent coupling, or the scaffold wall can be embedded or coated with growth factor-containing microspheres (Piotrowicz and Shoichet, 2006).

Each of the different methods for incorporating cargo into an engineered nerve conduit described above is associated with different release mechanisms and release kinetics. When a growth factor is simply in solution in the lumen of the conduit, release will occur via diffusion at a rate more rapid than desired for most applications. Incorporation of growth factor into degradable microspheres typically results in an initial burst release from the surface, followed by sustained release as the polymer breaks down and releases the encapsulated payload. Depending on the desired application, the release kinetics can be further tuned by modifying polymer composition, producing layered microspheres or conduits (Ahmed et al., 2012) or co-encapsulating a protein stabilizer, such as bovine serum albumin (BSA) (Coleman and Lowman, 2012). Piotrowicz and Shoichet investigated three different methods of incorporating NGF into a nerve conduit and quantifying release over a period of 28 days (Piotrowicz and Shoichet, 2006). A nerve conduit loaded by soaking in an NGF-containing solution released 95% (990 pg/cm) of the imbibed NGF by day 1, with only an additional 50 pg released through day 28. Embedding NGF-encapsulating microspheres into the inner wall of the conduit resulted in ∼30% burst release of NGF by day 1 and sustained release over the 28 day period, but only 220 pg/cm released overall. Optimal growth factor release was observed when NGF was directly entrapped within the inner wall of the conduit during synthesis. This method yielded the lowest burst release (∼23%) and the most consistent sustained release, with a total of 8624 pg/cm of released NGF during the study. This study demonstrates the differences in release kinetics that can be achieved with minor modifications to the fabrication of the nerve conduit.

The lack of an intact BNB can result in exposure of the nerve to undesirably high concentrations of delivered cargo. Basic strategies described above that deliver a single factor with rapid diffusion from the biomaterial scaffold can result in uncontrolled and aberrant axonal growth and improper innervation of peripheral targets (Liuzzi and Tedeschi, 1991). These issues can be due, in part, to poorly controlled release kinetics (including high burst release), difficulty of delivering the optimal dose *in vivo* (Kemp et al., 2011), and the administration of a single growth factor (Madduri et al., 2010) which may not properly capture the complex physiological milieu that is conducive to regeneration and repair. In response to these issues, recent strategies to engineer nerve conduits focus on more complex release kinetics by engineering systems to release several growth factors in a coordinated sequence or synergistically (Madduri et al., 2010; Zhang et al., 2014). For example, in a recent study the cytokine erythropoietin was incorporated into a NGF delivery system in a manner that facilitated their synergism through coordinated release (Zhang et al., 2017). The nerve conduit contained two different preparations of microspheres: erythropoietin was encapsulated in PLGA microspheres to allow for a burst release followed by sustained release, and NGF was encapsulated in BSA-containing PLGA microspheres which resulted in its delayed release. Dual delivery of the compounds resulted in a higher sciatic functional index and motor nerve conduction velocity both 4 and 8 weeks post-injury as compared to animals receiving either treatment alone. In addition, an increased number of myelinated axons and thicker bands of myelin were observed in animals receiving both preparations of microspheres after 8 weeks of treatment.

Another recent study utilized a core-shell layering strategy to deliver vascular endothelial growth factor (VEGF) followed by NGF (Xia and Lv, 2018). In this system, NGF was emulsified into the poly (l-lactic acid) (PLLA) core of an electrospun, nanofibrous scaffold. Following synthesis of the scaffold, a shell of VEGF was established by physical adsorption onto the surface. The nanofibrous scaffold provided a large surface area for adsorption of a maximal amount of VEGF, and ensured rapid, local release of the angiogenic factor. The initial burst release of VEGF promoted vascularization of the scaffold, and the sustained release of NGF promoted nerve regeneration. A rat sciatic nerve transection model revealed that this layered dual-delivery resulted in improved neovascularization and nerve healing as compared to either growth factor alone, with VEGF potentiating the ability of NGF to promote regeneration.

In addition to locally administering endogenous growth factors, engineered nerve conduits can be employed to deliver small molecules that may be intolerable at high systemic doses. Many of these compounds are approved at low doses for other applications, and have been identified for possible off-label use in research. For example, tacrolimus (FK506) is an immunosuppressant used in conjunction with cyclosporin A following organ transplantation that has several non-immune effects attributed to its use. Early studies demonstrated that FK506 promotes neurite outgrowth *in vitro* (Lyons et al., 1994) and enhances axonal regeneration *in vivo* (Gold et al., 1995). These effects are dose-dependent, and there are side-effects associated with high-dose or long-term administration (Yan et al., 2012). In Labroo et al. (2016) designed a double-walled PLGA nerve conduit that released active FK506 for several weeks and enhanced dorsal root ganglion (DRG) outgrowth *in vitro*. In a subsequent study, administration of FK506 along with NGF and/or GDNF exhibited a potentiating effect, with significantly enhanced nerve elongation and branching *in vitro* (Labroo et al., 2017). The future for FK506 in targeted treatment of peripheral nerve regeneration lies in more sophisticated drug delivery systems that facilitate co-administration *in vivo* along with the growth factors described above.

In the above described delivery systems, the presence of a breached barrier and surgical placement of a nerve graft allow for the use of a complex delivery systems that incorporate coordinated release of multiple, synergistic factors. Optimization of this approach is primarily focused on identifying the best compound, or combination of compounds, for delivery and determining optimal release kinetics. Here, the “targeting” mechanism is simple: direct placement at the nerve and unobstructed access to the endoneurium. This is not the case for delivery in the absence of injury.

## Strategy II: Diffusion Across an Intact Barrier

While injury and a breached barrier influence the bioengineering strategies described in the previous section, situations requiring administration of anesthesia or analgesia to peripheral nerves necessitate a different approach. In these cases, the perineurium and BNB are often intact at the desired site of administration, surgical administration is unnecessary or contraindicated, and the anatomy of the peripheral nerve becomes a key hurdle to overcome for delivery of compounds to affected axons (Vadhanan et al., 2015). Strategies for administration of peripheral nerve block or relief of neuropathic pain depend on the ability of a compound to diffuse freely across the perineurium and the BNB, which is related, in part, to its solubility. For the purposes of this review, we will discuss strategies to deliver both conventional local anesthetics and peptide or protein biologics to the endoneurium.

### Local Anesthetics

After peripheral injection, conventional local anesthetics can diffuse across the barriers of the PNS and access motor and sensory axons due to their hydrophobic nature. Nerve stimulation or ultrasound guidance enable precise localization of the injection with respect to peripheral nerve architecture: outside of the epineurium, outside of the perineurium, or within the perineurium (intraneural injection) (Figure 1). Nerve injury after peripheral nerve block can occur as a result of many factors, including toxicity, dose, volume, pressure, and blunt forces of the needle. Intraneural injection may lead to a longer lasting nerve block than extrafasicular injection (Damjanovska et al., 2015); however, local anesthetics can also cause apoptosis, injury, or long-term neurological deficits when injected in this manner (Farber et al., 2013; Brull et al., 2015). Sustained release formulations, such as liposomal bupivacaine, may decrease the incidence of systemic toxicity (Santamaria et al., 2017), but conflicting data surround the application of such formulations to minimize local toxicity (McAlvin et al., 2013; Damjanovska et al., 2015). While the occurrence of permanent nerve damage following peripheral nerve block is rare (Lirk et al., 2011), it remains difficult to balance desired properties of a nerve block (fast onset) with adverse effects (toxicity and transient nerve injury), particularly in patients with preexisting neurological disease (Lirk et al., 2011).

A strategy to advance peripheral anesthesia and pain control methods incorporates the use of stimulus-responsive biomaterials. Such a delivery system would release minimal drug in the absence of an external stimulus, allowing for on-demand adjustment of the local dose of anesthesia according to the patient’s needs. Several methods of external triggering can be engineered into delivery systems to facilitate on-demand delivery of a payload, including near infrared (NIR) light (Rwei et al., 2015) and ultrasound (Epstein-Barash et al., 2010).

Ultrasound has recently been investigated as a trigger for an on-demand system to release local anesthetic. An attractive feature of this system is that ultrasound is already incorporated into clinical practice to guide the placement of epi- and intraneural injections of local anesthetics (Marhofer et al., 2010). Rwei et al. (2017a) synthesized liposomes containing a sonosensitizer which, with the application of ultrasound energy, released reactive oxygen species that peroxidated the unsaturated lipid component of the liposome. This led to release of the encapsulated anesthetic, tetrodotoxin (TTX), and subsequent nerve block. When co-administered with an α2-adrenergic agonist, dexmedetomidine, the duration of the nerve block could be controlled by varying the duration of the applied insonation.

The same investigators have also explored local anesthetic delivery systems that are triggered by near-infrared light (Rwei et al., 2015, 2017b). In this case, the liposomal formulation contains a NIR-absorbing photosensitizer that triggers release of TTX in the presence of 730 nm light. While the system produced a nerve block with distinct on-off states, NIR light does not penetrate tissue to the depth that ultrasound does, and is not a component of the standard of care, which may make NIR-triggered systems less desirable than ultrasound mediated delivery. Incorporation of dexmedetomidine may potentiate the effect of TTX, facilitating release with decreased intensity and duration of irradiation (Rwei et al., 2017a).

### Hydrophilic Compounds

An alternative to the application of potentially toxic local anesthetics for the management of pain or production of sustained nerve block is the use of endogenous, hydrophilic opioid peptides. Mu-opioid receptors are expressed along the axons of peripheral nerves and are upregulated after injury or inflammation (Truong et al., 2003; Schmidt et al., 2013; Mousa et al., 2017). While mu-opioid receptors are expressed and functional in non-injured conditions (Mambretti et al., 2016), they are quiescent, as their ligands cannot breach the intact BNB and access the axons of sensory neurons. Thus, the local application of opioids as a pain management strategy requires targeted disruption of the BNB.

Several strategies exist to disrupt the perineural and endoneurial barriers and facilitate targeted delivery of hydrophilic compounds. One strategy extends earlier findings that transient opening of the blood-brain barrier can be achieved through infusion of a hypertonic solution (Rapoport, 2000). Initial studies with peripheral nociception demonstrated that plantar injection of hypertonic saline resulted in sustained but transient BNB opening and potent peripheral antinociception after plantar administration of the endogenous opioids met-enkephalin and β-endorphin (Rittner et al., 2009). Subsequent studies incorporating small interfering RNA (siRNA) knockdown of components of peripheral nerve tight junctions demonstrated that perisciatic injection of hypertonic saline opens the BNB by a mechanism involving reduced expression of claudin-1, but not claudin-5 or occludin tight junction proteins. Perineural administration of siRNA to claudin-1 facilitated local delivery of hydrophilic opioids and subsequent nociception (Hackel et al., 2012).

Following the determination that the barrier-opening effects of hypertonic saline were mediated through claudin-1, subsequent studies demonstrated that perineural application of a claudin-1 peptidomimetic facilitated barrier opening and transport of hydrophilic analgesics to axonal nociceptors in normal rats (Zwanziger et al., 2012; Sauer et al., 2014; Staat et al., 2015). Further, perineural application of recombinant tissue plasminogen activator has similarly been demonstrated to downregulate claudin-1 expression and increase permeability of the BNB via a separate mechanism which is, in part, regulated by specific microRNAs (Yang et al., 2016). In summary, these studies elegantly identify several mechanistic pathways that can be targeted to transiently open the BNB to facilitate delivery of hydrophilic small molecules or compounds to peripheral nerves. Future studies may incorporate these barrier-opening strategies into on-demand delivery systems to clinically advance patient-centered pain treatment.

## Strategy III: Retrograde Axonal Transport, Circumventing the Barrier

One strategy that confers targeted delivery to the PNS despite the presence of an intact BNB employs basic principles of gene therapy. Initial approaches to nerve-targeted gene therapy exploited the natural neurotropism exhibited by many viral vectors, such as the affinity of herpes simplex virus for peripheral sensory neurons of the DRG (Wolfe et al., 2012). While results have been promising, viral gene delivery is not ideal for *in vivo* or clinical application due to toxic, inflammatory, and immunogenic complications and safety concerns.

Several natural toxins exist that bind to receptors located within the central and peripheral nervous systems and could be used to engineer a targeted delivery system (Lencer and Tsai, 2003; Surana et al., 2018). Targeted uptake by presynaptic peripheral nerve terminals can be achieved with bacterial neurotoxins, either with a cell-penetrating peptide derived from the toxin (Liu et al., 2005) or an inactive fragment of holotoxin (Lopes et al., 2017). While this component conveys target specificity to the PNS, incorporation of synthetic (Lopes et al., 2016) or naturally occurring (Oliveira et al., 2010) biomaterials into the delivery system facilitates the formation of nanocomplexes which will protect delivery cargo (e.g., nucleic acids) from degradation as well as facilitate cell-specific uptake. In this manner, intramuscularly injected cargo can be delivered to peripheral nerves in the presence of an intact BNB, exploiting natural retrograde axonal transport mechanisms employed by toxins.

Tetanus toxins are clostridial toxins that exhibit high affinity binding to ganglioside receptors, primarily the ganglioside GT1b receptor, on the presynaptic terminals of peripheral nerves (Surana et al., 2018). This high affinity binding conveys extreme potency, with lethal doses as low as 0.1 ng/kg of body weight. The primary mechanism by which tetanus toxin exerts effects on the PNS involves efficient retrograde transport from the neuromuscular junction to the cell body of motor neurons; however, it is also taken up and transported by sensory and sympathetic neurons (Wilson and Ho, 2014; Surana et al., 2018). The C-terminal binding domain of tetanus toxin has been demonstrated to mediate the effects of the holotoxin (Surana et al., 2018). Inactive mutants of, or peptides derived from, the C-terminal binding domain have been successfully engineered to target cargo to the peripheral nerves.

Tet1, a 12-amino acid peptide, was identified with phage display as exhibiting both an affinity for ganglioside GT1b and binding properties similar to the C-terminal binding domain of tetanus toxin (Liu et al., 2005). Subsequent studies demonstrated the *in vitro* (Federici et al., 2007) and *in vivo* uptake and retrograde transport of Tet1-conjugated green fluorescent protein (GFP) to the motor neuron cell body from the site of injection (*lumborum of latissimus dorsi*) (Kassa et al., 2013). To test the delivery of nucleic acids in a targeted system, Chu et al. (2013) prepared copolymers of *N*-(2-hydroxypropyl) methacrylamide (HPMA) and oligolysine/Tet1 macromonomers that formed complexes with plasmid DNA. In this system, Tet1 serves as the neuron-specific targeting entity, oligolysine serves to bind and protect nucleic acid, and HPMA provides stability. The authors demonstrated successful gene delivery to neurons *in vitro* (Chu et al., 2013). Complexes of Tet1 and polyethyleneimine (PEI), as well as polymerosomes composed of poly(*E*-caprolactone)-*block*-poly(ethylene glycol) (PEG-*b*-PCL) have also been demonstrated to deliver nucleic acids to central nervous system (CNS) neurons when utilizing Tet1 as a targeting moiety (Park et al., 2007; Zhang et al., 2012).

While Tet1 has been incorporated as a targeting moiety into neuron-specific delivery systems, further advances have been made with inactive, nontoxic mutants or fragments of the tetanus toxin holoenzyme. Lopes et al., 2016 prepared PEI-based nanoparticles functionalized with PEGylated nontoxic c-terminal fragments of tetanus toxin. The PEI formed complexes with DNA encoding GFP and β-galactosidase. Noninvasive injection into the footpad resulted in GFP expression in DRG cell bodies three days later, with minimal expression in non-neuronal tissues.

More recently, the investigators employed a similar delivery system to investigate delivery of a neurotrophic factor in a clinically relevant *in vivo* nerve crush injury model (Lopes et al., 2017). Instead of PEI, which can be toxic, the biodegradable cationic polymer chitosan was used to form nanocomplexes with DNA encoding BDNF. Nanocomplexes were then conjugated with PEGylated c-terminal fragments of tetanus toxin, forming nanoparticles. Animals received an intramuscular (gastrocnemius) injection of nanoparticles eight days prior to nerve crush injury. Delivery of BDNF plasmid DNA through this chitosan-based vector resulted in increased BDNF protein expression in DRG, lumbar spinal cord, and sciatic nerve at the end of the 21-day study, as well as significant sensorimotor functional recovery. This marks one of the first studies to demonstrate successful delivery of a neurotrophic gene selectively to peripheral neurons, leading to functional recovery in a nerve injury model.

Botulinum toxin is another member of the clostridium neurotoxin family that can target delivery to the peripheral nerves in the presence of an intact BNB following a noninvasive peripheral injection. Botulinum toxin is taken up by motor neurons where it blocks acetylcholine release at the neuromuscular junction (Surana et al., 2018). While it was initially thought that all isoforms of botulinum toxin remained at the nerve terminal, it has been established that a particular isoform, botulinum neurotoxin A (BoNT/A), is subject to retrograde trafficking, making it a candidate for targeting to the soma (Restani et al., 2012).

Retrograde trafficking of BoNT/A was investigated *in vivo* in a mouse model of neuropathic pain (Marinelli et al., 2012). Animals received a noninvasive intraplantar injection of BoNT/A five days after chronic constriction injury. The dose of BoNT/A was such that paralysis was not induced. Botulinum activity was detected in the peripheral nerve endings, along the axons, and in DRG, as well as in spinal cord and spinal astrocytes. Further, animals displayed significant reduction in mechanical allodynia as well as an increase in functional recovery, suggesting that botulinum toxin may exert central effects beyond cholinergic synapse modulation that result in analgesia and attenuation of pain pathways (Pavone and Luvisetto, 2010). A recent study by the same investigators (Finocchiaro et al., 2018) studied the effects of BoNT/B isoform on mechanical allodynia and functional recovery. They observed analgesic effects, but not functional recovery, highlighting differences between the subtypes of botulinum isoforms that may have implications in the design of targeting strategies.

Cholera toxin has an atoxic subunit (cholera toxin subunit B, or CTB) which displays a natural neurotropism through its binding to the ganglioside GM1. The specificity of CTB for GM1 has recently been exploited to target mesoporous silicone nanoparticles (MSNPs) to motor neurons (Gonzalez Porras et al., 2016). This system improves upon the cationic complexes described above by allowing for a larger payload capacity within the cylindrical pores of the nanoparticle. After loading with a model small molecule drug, MSNPs were coated with a preparation of phospholipids and cholesterol, and biotin-CTB was conjugated to the surface of the nanoparticles. CTB functionalized MSNPs displayed motoneuron cell specificity and delivered a small molecule to motoneurons *in vitro*, while remaining nontoxic. Recently, the authors tested the application of MSNPs *in vivo* (Gonzalez Porras et al., 2018), and demonstrated the presence of fluorescent CTB functionalized MSNPs in the phrenic nerve 24h after intrapleural injection. The particles were taken up by endocytosis at lipid rafts, and evaded lysosomal degradation once inside the cell.

The next wave of neuron-targeted delivery strategies that exploit mechanisms of retrograde trafficking lies in delivery of diverse cargo that facilitates functional improvement in different disease models. In this regard, the payload capacity of mesoporous silicon nanoparticles may serve as a distinct advantage over cationic nanocomplexes, enabling the delivery of small molecules and proteins along the axon. However, as demonstrated by Lopes et al., delivery of plasmid DNA encoding a neurotrophic factor can result in increased protein expression and functional protection *in vivo*. Future successful delivery systems may combine aspects of each strategy to facilitate coordinated release of multiple factors. The emergence of these studies in just the past year or two suggests that this field will be rapidly expanding.

## Strategy IV: Endothelial Targeting From the Systemic Circulation

Despite advances in bioengineering to deliver compounds to the peripheral nerve as described above, targeted delivery for treatment of diseases of the PNS remain elusive. The sheer length of peripheral nerves may necessitate systemic delivery, while the selectivity of the BNB restricts access of systemically administered agents to the cells of the PNS. The most promising strategies will incorporate PNS-specific modifications to systemically administered nanocarriers, such that the administered compounds will (I) be protected from degradation/phagocytosis/opsonization within the circulation, (II) identify and recognize the targeted peripheral nerves, and (III) be able to cross the BNB.

A novel preparation of dioleoyl-phosphatidylcholine (DOPC) based liposomes preferentially targeted peripheral neurons and Schwann cells when conferred with some key modifications that mimic properties of neural cell membranes. Investigators added Poloxamer 188, a nonionic emulsifier that has been demonstrated to facilitate transport across the blood brain barrier, and supplemented liposomes with cholesterol, which is expressed at high levels in neuronal cells, especially myelinating glia (Lee et al., 2013). *In vitro* experiments demonstrated that fluorescently labeled DOPC liposomes were internalized by Schwann cells, sensory neurons, and motor axons, but not skeletal muscle cells. When administered intravenously, DOPC liposomes were detected at elevated levels in sciatic nerve Schwann cells as well as in highly vascularized regions of the brain. Detection of DOPC liposomes in the liver and kidney indicated clearance through these routes; however, collectively the data suggest that modified liposomes do cross the BNB and are taken up by myelinated peripheral nerves.

While liposomal preparations may confer specificity to neurons, it may be necessary for a delivery system to target PNS-specific receptors expressed by BNB endothelial cells, such that they may be accessed from the circulation. Several strategies have demonstrated delivery of cargo across the blood-brain barrier by targeting CNS-specific endothelial receptors, a situation analogous to that encountered in the periphery. The localized expression pattern of transporters and receptors to brain endothelial cells facilitates carrier-mediated transport of circulating particles with a surface modified to express the ligand (Thuenauer et al., 2017). Delivery across the blood-brain barrier has been successful with nanoparticles functionalized to express apolipoprotein E (Dal Magro et al., 2017) or angiopep-2 peptide (Wang et al., 2015), which both target upregulated low-density lipoprotein receptors on brain endothelial cells, or with nanoparticles modified to express a monoclonal antibody to the transferrin receptor (Wiley et al., 2013).

The unique molecular composition of transporters, receptors, and tight junction associated molecules that make up the BNB are not fully defined. Much of the current knowledge of the BNB is derived from *in vitro* cultures (Ubogu, 2013), and, more recently, *in situ* observations (Palladino et al., 2017). The glucose transporter GLUT-1 is highly expressed in perineural and endoneurial endothelial cells, while endoneurial endothelial cells also express the creatine (CRT) and monocarboxylic acid (MCT-1) nutrient transporters (Yosef and Ubogu, 2013). The cell membrane localization of these transporters and potential for use as a PNS targeting mechanism remains to be elucidated.

Inflammatory neuropathies are characterized by upregulated expression of cell adhesion molecules and other inflammatory markers that may be exploited to target therapies to the inflamed peripheral nerves. For example, localized expression of intercellular adhesion molecule (ICAM)-1 by endoneurial endothelial cells during inflammation has been observed *in vitro* (Langert et al., 2013) and *in vivo* (Putzu et al., 2000). Nanoparticles have been engineered to express a peptide fragment of leukocyte function antigen (LFA)-1, the high affinity ligand for ICAM-1 (Zhang et al., 2008). LFA-1-peptide functionalized PLGA nanoparticles were used to deliver the chemotherapeutic drug, doxorubicin, to ICAM-1-expressing carcinomic alveolar epithelial cells *in vitro* (Chittasupho et al., 2009). Similarly, nanoparticles coated with a monoclonal antibody to ICAM-1 bound to and were internalized by brain microvascular endothelial cells (Hsu et al., 2014). While *in vivo* targeting of adhesion molecules is just beginning to be explored for other applications (Khodabandehlou et al., 2017), ICAM-1 targeted nanoparticles may represent a novel means of targeting the inflamed PNS from the systemic circulation.

## Conclusion

Bioengineering has fueled advances in delivery of therapeutic cargo to the PNS, which can be difficult due to the presence of the BNB. The strategies that will be incorporated by a drug delivery system are dictated by the status of the BNB during injury, disease, or homeostatic conditions. These diverse strategies include coordinated release of multiple growth factors at the site of injury to facilitate repair in the presence of a breached barrier, or transient, localized barrier permeabilization and on-demand release to deliver peripheral nerve anesthesia or analgesia. In addition, the neurotropism of certain bacterial toxins can be exploited to deliver a customizable array of encapsulated cargo to the peripheral nerve via retrograde axonal transport for treatment of inherited neuropathies, neuropathic pain, and nerve injury. Finally, methods of targeting the BNB from the systemic circulation may include specific formulations of liposomes and surface functionalization with endothelial ligands. While much progress has been made, future clinical applications of targeted delivery to the peripheral nerve rely on continued advancement of biomaterials-based drug delivery systems, as well as further elucidation of the molecular characteristics of the BNB during health and disease.

## Author Contributions

KL conceptualized the topic and prepared the first draft of the article. EB revised all sections critically. Both authors read and approved the final submitted version.

## Conflict of Interest Statement

The authors declare that the research was conducted in the absence of any commercial or financial relationships that could be construed as a potential conflict of interest.

## References

[B1] Abram S. E. Yi J. Fuchs A. Hogan Q. H. (2006). Permeability of injured and intact peripheral nerves and dorsal root ganglia. *Anesthesiology* 105 146–153. 10.1097/00000542-200607000-00024 16810006

[B2] Ahmed A. R. Elkharraz K. Irfan M. Bodmeier R. (2012). Reduction in burst release after coating poly(D,L-lactide-co-glycolide) (PLGA) microparticles with a drug-free PLGA layer. *Pharm. Dev. Technol.* 17 66–72. 10.3109/10837450.2010.513989 20854130

[B3] Black K. A. Lin B. F. Wonder E. A. Desai S. S. Chung E. J. Ulery B. D. (2015). Biocompatibility and characterization of a peptide amphiphile hydrogel for applications in peripheral nerve regeneration. *Tissue Eng. Part A* 21 1333–1342. 10.1089/ten.tea.2014.0297 25626921 PMC4394881

[B4] Brull R. Hadzic A. Reina M. A. Barrington M. J. (2015). Pathophysiology and etiology of nerve injury following peripheral nerve blockade. *Reg. Anesth. Pain Med.* 40 479–490. 10.1097/AAP.0000000000000125 25974275

[B5] Catrina S. Gander B. Madduri S. (2013). Nerve conduit scaffolds for discrete delivery of two neurotrophic factors. *Eur. J. Pharm. Biopharm.* 85 139–142. 10.1016/j.ejpb.2013.03.030 23958324

[B6] Chittasupho C. Xie S. X. Baoum A. Yakovleva T. Siahaan T. J. Berkland C. J. (2009). ICAM-1 targeting of doxorubicin-loaded PLGA nanoparticles to lung epithelial cells. *Eur. J. Pharm. Sci.* 37 141–150. 10.1016/j.ejps.2009.02.008 19429421 PMC2778606

[B7] Chu D. S. Schellinger J. G. Bocek M. J. Johnson R. N. Pun S. H. (2013). Optimization of Tet1 ligand density in HPMA-co-oligolysine copolymers for targeted neuronal gene delivery. *Biomaterials* 34 9632–9637. 10.1016/j.biomaterials.2013.08.045 24041424 PMC3855292

[B8] Coleman J. Lowman A. (2012). Biodegradable nanoparticles for protein delivery: analysis of preparation conditions on particle morphology and protein loading, activity and sustained release properties. *J. Biomater. Sci. Polym. Ed.* 23 1129–1151. 10.1163/092050611X576648 21639993

[B9] Dal Magro R. Ornaghi F. Cambianica I. Beretta S. Re F. Musicanti C. (2017). ApoE-modified solid lipid nanoparticles: a feasible strategy to cross the blood-brain barrier. *J. Control. Release* 249 103–110. 10.1016/j.jconrel.2017.01.039 28153761

[B10] Damjanovska M. Cvetko E. Hadzic A. Seliskar A. Plavec T. Mis K. (2015). Neurotoxicity of perineural vs intraneural-extrafascicular injection of liposomal bupivacaine in the porcine model of sciatic nerve block. *Anaesthesia* 70 1418–1426. 10.1111/anae.13189 26338496 PMC5049634

[B11] de Boer R. Borntraeger A. Knight A. M. Hebert-Blouin M. N. Spinner R. J. Malessy M. J. (2012). Short- and long-term peripheral nerve regeneration using a poly-lactic-co-glycolic-acid scaffold containing nerve growth factor and glial cell line-derived neurotrophic factor releasing microspheres. *J. Biomed. Mater. Res. A* 100 2139–2146. 10.1002/jbm.a.34088 22615148

[B12] Dinis T. M. Elia R. Vidal G. Dermigny Q. Denoeud C. Kaplan D. L. (2015). 3D multi-channel bi-functionalized silk electrospun conduits for peripheral nerve regeneration. *J. Mech. Behav. Biomed. Mater.* 41 43–55. 10.1016/j.jmbbm.2014.09.029 25460402

[B13] Epstein-Barash H. Orbey G. Polat B. E. Ewoldt R. H. Feshitan J. Langer R. (2010). A microcomposite hydrogel for repeated on-demand ultrasound-triggered drug delivery. *Biomaterials* 31 5208–5217. 10.1016/j.biomaterials.2010.03.008 20347484 PMC3072837

[B14] Farber S. J. Saheb-Al-Zamani M. Zieske L. Laurido-Soto O. Bery A. Hunter D. (2013). Peripheral nerve injury after local anesthetic injection. *Anesth. Analg.* 117 731–739. 10.1213/ANE.0b013e3182a00767 23921658

[B15] Federici T. Liu J. K. Teng Q. Yang J. Boulis N. M. (2007). A means for targeting therapeutics to peripheral nervous system neurons with axonal damage. *Neurosurgery* 60 911–918; discussion 911–918. 10.1227/01.NEU.0000255444.44365.B9 17460527

[B16] Finocchiaro A. Marinelli S. De Angelis F. Vacca V. Luvisetto S. Pavone F. (2018). Botulinum toxin B affects neuropathic pain but not functional recovery after peripheral nerve injury in a mouse model. *Toxins (Basel)* 10:E128. 10.3390/toxins10030128 29562640 PMC5869416

[B17] Fu K. Y. Dai L. G. Chiu I. M. Chen J. R. Hsu S. H. (2011). Sciatic nerve regeneration by microporous nerve conduits seeded with glial cell line-derived neurotrophic factor or brain-derived neurotrophic factor gene transfected neural stem cells. *Artif. Organs* 35 363–372. 10.1111/j.1525-1594.2010.01105.x 21314831

[B18] Gold B. G. Katoh K. Storm-Dickerson T. (1995). The immunosuppressant FK506 increases the rate of axonal regeneration in rat sciatic nerve. *J. Neurosci.* 15 7509–7516. 10.1523/JNEUROSCI.15-11-07509.1995 7472502 PMC6578050

[B19] Gonzalez Porras M. A. Durfee P. Giambini S. Sieck G. C. Brinker C. J. Mantilla C. B. (2018). Uptake and intracellular fate of cholera toxin subunit b-modified mesoporous silica nanoparticle-supported lipid bilayers (aka protocells) in motoneurons. *Nanomedicine* 14 661–672. 10.1016/j.nano.2018.01.002 29339186 PMC7754615

[B20] Gonzalez Porras M. A. Durfee P. N. Gregory A. M. Sieck G. C. Brinker C. J. Mantilla C. B. (2016). A novel approach for targeted delivery to motoneurons using cholera toxin-B modified protocells. *J. Neurosci. Methods* 273 160–174. 10.1016/j.jneumeth.2016.09.003 27641118 PMC5574179

[B21] Hackel D. Krug S. M. Sauer R. S. Mousa S. A. Bocker A. Pflucke D. (2012). Transient opening of the perineurial barrier for analgesic drug delivery. *Proc. Natl. Acad. Sci. U.S.A.* 109 E2018–E2027. 10.1073/pnas.1120800109 22733753 PMC3406837

[B22] Han F. Luo B. Shi R. Han C. Zhang Z. Xiong J. (2014). Curcumin ameliorates rat experimental autoimmune neuritis. *J. Neurosci. Res.* 92 743–750. 10.1002/jnr.23357 24482305

[B23] Hsu J. Rappaport J. Muro S. (2014). Specific binding, uptake, and transport of ICAM-1-targeted nanocarriers across endothelial and subendothelial cell components of the blood-brain barrier. *Pharm. Res.* 31 1855–1866. 10.1007/s11095-013-1289-8 24558007 PMC4065215

[B24] Hsu S. H. Kuo W. C. Chen Y. T. Yen C. T. Chen Y. F. Chen K. S. (2013). New nerve regeneration strategy combining laminin-coated chitosan conduits and stem cell therapy. *Acta Biomater.* 9 6606–6615. 10.1016/j.actbio.2013.01.025 23376237

[B25] Huang Y. C. Hsu S. H. Kuo W. C. Chang-Chien C. L. Cheng H. Huang Y. Y. (2011). Effects of laminin-coated carbon nanotube/chitosan fibers on guided neurite growth. *J. Biomed. Mater. Res. A* 99 86–93. 10.1002/jbm.a.33164 21800418

[B26] Kanda T. (2013). Biology of the blood-nerve barrier and its alteration in immune mediated neuropathies. *J. Neurol. Neurosurg. Psychiatry* 84 208–212. 10.1136/jnnp-2012-302312 23243216

[B27] Kassa R. Monterroso V. David L. L. Tshala-Katumbay D. (2013). Diagnostic and therapeutic potential of tetanus toxin-derivatives in neurological diseases. *J. Mol. Neurosci.* 51 788–791. 10.1007/s12031-013-0065-x 23842888 PMC3838929

[B28] Kemp S. W. Webb A. A. Dhaliwal S. Syed S. Walsh S. K. Midha R. (2011). Dose and duration of nerve growth factor (NGF) administration determine the extent of behavioral recovery following peripheral nerve injury in the rat. *Exp. Neurol.* 229 460–470. 10.1016/j.expneurol.2011.03.017 21458449

[B29] Khodabandehlou K. Masehi-Lano J. J. Poon C. Wang J. Chung E. J. (2017). Targeting cell adhesion molecules with nanoparticles using in vivo and flow-based in vitro models of atherosclerosis. *Exp. Biol. Med. (Maywood)* 242 799–812. 10.1177/1535370217693116 28195515 PMC5407539

[B30] Labroo P. Ho S. Sant H. Shea J. Gale B. K. Agarwal J. (2016). Controlled delivery of FK506 to improve nerve regeneration. *Shock* 46(3 Suppl. 1),154–159. 10.1097/SHK.0000000000000628 27058050

[B31] Labroo P. Shea J. Sant H. Gale B. Agarwal J. (2017). Effect of combining FK506 and neurotrophins on neurite branching and elongation. *Muscle Nerve* 55 570–581. 10.1002/mus.25370 27503321 PMC5517102

[B32] Langert K. A. Goshu B. Stubbs E. B. Jr. (2017). Attenuation of experimental autoimmune neuritis with locally administered lovastatin-encapsulating poly(lactic-co-glycolic) acid nanoparticles. *J. Neurochem.* 140 334–346. 10.1111/jnc.13892 27861905 PMC5225029

[B33] Langert K. A. Von Zee C. L. Stubbs E. B. (2013). Tumour necrosis factor alpha enhances CCL2 and ICAM-1 expression in peripheral nerve microvascular endoneurial endothelial cells. *ASN Neuro* 5:art:e00104. 10.1042/AN20120048 23293927 PMC3565377

[B34] Lee A. C. Yu V. M. Lowe J. B. III Brenner M. J. Hunter D. A. Mackinnon S. E. (2003). Controlled release of nerve growth factor enhances sciatic nerve regeneration. *Exp. Neurol.* 184 295–303. 10.1016/S0014-4886(03)00258-9 14637100

[B35] Lee S. Ashizawa A. T. Kim K. S. Falk D. J. Notterpek L. (2013). Liposomes to target peripheral neurons and Schwann cells. *PLoS One* 8:e78724. 10.1371/journal.pone.0078724 24244347 PMC3823803

[B36] Lencer W. I. Tsai B. (2003). The intracellular voyage of cholera toxin: going retro. *Trends Biochem. Sci.* 28 639–645. 10.1016/j.tibs.2003.10.002 14659695

[B37] Liodaki E. Bos I. Lohmeyer J. A. Senyaman O. Mauss K. L. Siemers F. (2013). Removal of collagen nerve conduits (NeuraGen) after unsuccessful implantation: focus on histological findings. *J. Reconstr. Microsurg.* 29 517–522. 10.1055/s-0033-1348033 23818251

[B38] Lirk P. Birmingham B. Hogan Q. (2011). Regional anesthesia in patients with preexisting neuropathy. *Int. Anesthesiol. Clin.* 49 144–165. 10.1097/AIA.0b013e3182101134 21956084

[B39] Liu H. Chen Y. Huang L. Sun X. Fu T. Wu S. (2018). Drug distribution into peripheral nerve. *J. Pharmacol. Exp. Ther.* 365 336–345. 10.1124/jpet.117.245613 29511033

[B40] Liu J. K. Teng Q. Garrity-Moses M. Federici T. Tanase D. Imperiale M. J. (2005). A novel peptide defined through phage display for therapeutic protein and vector neuronal targeting. *Neurobiol. Dis.* 19 407–418. 10.1016/j.nbd.2005.01.022 16023583

[B41] Liuzzi F. J. Tedeschi B. (1991). Peripheral nerve regeneration. *Neurosurg. Clin. N. Am.* 2 31–42. 10.1016/S1042-3680(18)30755-1 1821734

[B42] Lopes C. D. Oliveira H. Estevao I. Pires L. R. Pego A. P. (2016). In vivo targeted gene delivery to peripheral neurons mediated by neurotropic poly(ethylene imine)-based nanoparticles. *Int. J. Nanomed.* 11 2675–2683. 10.2147/IJN.S104374 PMC4907712 27354797

[B43] Lopes C. D. F. Goncalves N. P. Gomes C. P. Saraiva M. J. Pego A. P. (2017). BDNF gene delivery mediated by neuron-targeted nanoparticles is neuroprotective in peripheral nerve injury. *Biomaterials* 121 83–96. 10.1016/j.biomaterials.2016.12.025 28081461

[B44] Lyons W. E. George E. B. Dawson T. M. Steiner J. P. Snyder S. H. (1994). Immunosuppressant FK506 promotes neurite outgrowth in cultures of PC12 cells and sensory ganglia. *Proc. Natl. Acad. Sci. U.S.A.* 91 3191–3195. 10.1073/pnas.91.8.3191 7512727 PMC43541

[B45] Madduri S. di Summa P. Papaloizos M. Kalbermatten D. Gander B. (2010). Effect of controlled co-delivery of synergistic neurotrophic factors on early nerve regeneration in rats. *Biomaterials* 31 8402–8409. 10.1016/j.biomaterials.2010.07.052 20692034

[B46] Madduri S. Gander B. (2012). Growth factor delivery systems and repair strategies for damaged peripheral nerves. *J. Control. Release* 161 274–282. 10.1016/j.jconrel.2011.11.036 22178593

[B47] Mambretti E. M. Kistner K. Mayer S. Massotte D. Kieffer B. L. Hoffmann C. (2016). Functional and structural characterization of axonal opioid receptors as targets for analgesia. *Mol. Pain* 12:1744806916628734. 10.1177/1744806916628734 27030709 PMC4994859

[B48] Marhofer P. Harrop-Griffiths W. Kettner S. C. Kirchmair L. (2010). Fifteen years of ultrasound guidance in regional anaesthesia: part 1. *Br. J. Anaesth.* 104 538–546. 10.1093/bja/aeq069 20364022

[B49] Marinelli S. Vacca V. Ricordy R. Uggenti C. Tata A. M. Luvisetto S. (2012). The analgesic effect on neuropathic pain of retrogradely transported botulinum neurotoxin A involves Schwann cells and astrocytes. *PLoS One* 7:e47977. 10.1371/journal.pone.0047977 23110146 PMC3480491

[B50] McAlvin J. B. Reznor G. Shankarappa S. A. Stefanescu C. F. Kohane D. S. (2013). Local toxicity from local anesthetic polymeric microparticles. *Anesth. Analg.* 116 794–803. 10.1213/ANE.0b013e31828174a7 23460564 PMC3606664

[B51] Moreau N. Mauborgne A. Bourgoin S. Couraud P. O. Romero I. A. Weksler B. B. (2016). Early alterations of Hedgehog signaling pathway in vascular endothelial cells after peripheral nerve injury elicit blood-nerve barrier disruption, nerve inflammation, and neuropathic pain development. *Pain* 157 827–839. 10.1097/j.pain.0000000000000444 26655733

[B52] Mousa S. A. Shaqura M. Al-Madol M. Tafelski S. Khalefa B. I. Shakibaei M. (2017). Accessibility of axonal G protein coupled mu-opioid receptors requires conceptual changes of axonal membrane targeting for pain modulation. *J. Control. Release* 268 352–363. 10.1016/j.jconrel.2017.10.016 29054370

[B53] Nishihara H. Maeda T. Sano Y. Ueno M. Okamoto N. Takeshita Y. (2018). Fingolimod promotes blood-nerve barrier properties in vitro. *Brain Behav.* 8:e00924. 10.1002/brb3.924 29670818 PMC5893339

[B54] Oliveira H. Pires L. R. Fernandez R. Martins M. C. Simoes S. Pego A. P. (2010). Chitosan-based gene delivery vectors targeted to the peripheral nervous system. *J. Biomed. Mater. Res. A* 95 801–810. 10.1002/jbm.a.32874 20734332

[B55] Palladino S. P. Helton E. S. Jain P. Dong C. Crowley M. R. Crossman D. K. (2017). The human blood-nerve barrier transcriptome. *Sci. Rep.* 7:17477. 10.1038/s41598-017-17475-y 29234067 PMC5727190

[B56] Park I. K. Lasiene J. Chou S. H. Horner P. J. Pun S. H. (2007). Neuron-specific delivery of nucleic acids mediated by Tet1-modified poly(ethylenimine). *J. Gene Med.* 9 691–702. 10.1002/jgm.1062 17582226 PMC2633605

[B57] Pavone F. Luvisetto S. (2010). Botulinum neurotoxin for pain management: insights from animal models. *Toxins (Basel)* 2 2890–2913. 10.3390/toxins2122890 22069581 PMC3153188

[B58] Peltonen S. Alanne M. Peltonen J. (2013). Barriers of the peripheral nerve. *Tissue Barriers* 1:e24956. 10.4161/tisb.24956 24665400 PMC3867511

[B59] Piotrowicz A. Shoichet M. S. (2006). Nerve guidance channels as drug delivery vehicles. *Biomaterials* 27 2018–2027. 10.1016/j.biomaterials.2005.09.042 16239029

[B60] Pottabathini R. Kumar A. Bhatnagar A. Garg S. Ekavali E. (2016). Ameliorative potential of pioglitazone and ceftriaxone alone and in combination in rat model of neuropathic pain: targeting PPARgamma and GLT-1 pathways. *Pharmacol. Rep.* 68 85–94. 10.1016/j.pharep.2015.06.010 26721358

[B61] Putzu G. A. Figarella-Branger D. Bouvier-Labit C. Liprandi A. Bianco N. Pellissier J. F. (2000). Immunohistochemical localization of cytokines, C5b-9 and ICAM-1 in peripheral nerve of Guillain-Barre syndrome. *J. Neurol. Sci.* 174 16–21. 10.1016/S0022-510X(99)00328-7 10704976

[B62] Quigley A. F. Bulluss K. J. Kyratzis I. L. Gilmore K. Mysore T. Schirmer K. S. (2013). Engineering a multimodal nerve conduit for repair of injured peripheral nerve. *J. Neural Eng.* 10:016008. 10.1088/1741-2560/10/1/016008 23283383

[B63] Rapoport S. I. (2000). Osmotic opening of the blood-brain barrier: principles, mechanism, and therapeutic applications. *Cell. Mol. Neurobiol.* 20 217–230. 10.1023/A:1007049806660 10696511 PMC11537517

[B64] Rechthand E. Smith Q. R. Rapoport S. I. (1987). Transfer of nonelectrolytes from blood into peripheral nerve endoneurium. *Am. J. Physiol.* 252(6 Pt 2), H1175–H1182. 10.1152/ajpheart.1987.252.6.H1175 3109260

[B65] Reid A. J. de Luca A. C. Faroni A. Downes S. Sun M. Terenghi G. (2013). Long term peripheral nerve regeneration using a novel PCL nerve conduit. *Neurosci. Lett.* 544 125–130. 10.1016/j.neulet.2013.04.001 23583695

[B66] Restani L. Giribaldi F. Manich M. Bercsenyi K. Menendez G. Rossetto O. (2012). Botulinum neurotoxins A and E undergo retrograde axonal transport in primary motor neurons. *PLoS Pathog.* 8:e1003087. 10.1371/journal.ppat.1003087 23300443 PMC3531519

[B67] Rittner H. L. Hackel D. Yamdeu R. S. Mousa S. A. Stein C. Schafer M. (2009). Antinociception by neutrophil-derived opioid peptides in noninflamed tissue-role of hypertonicity and the perineurium. *Brain Behav. Immun.* 23 548–557. 10.1016/j.bbi.2009.02.007 19233260

[B68] Rwei A. Y. Lee J. J. Zhan C. Liu Q. Ok M. T. Shankarappa S. A. (2015). Repeatable and adjustable on-demand sciatic nerve block with phototriggerable liposomes. *Proc. Natl. Acad. Sci. U.S.A.* 112 15719–15724. 10.1073/pnas.1518791112 26644576 PMC4697387

[B69] Rwei A. Y. Paris J. L. Wang B. Wang W. Axon C. D. Vallet-Regi M. (2017a). Ultrasound-triggered local anaesthesia. *Nat. Biomed. Eng.* 1 644–653. 10.1038/s41551-017-0117-6 29152410 PMC5687284

[B70] Rwei A. Y. Zhan C. Wang B. Kohane D. S. (2017b). Multiply repeatable and adjustable on-demand phototriggered local anesthesia. *J. Control. Release* 251 68–74. 10.1016/j.jconrel.2017.01.031 28153763 PMC5394744

[B71] Santamaria C. M. Woodruff A. Yang R. Kohane D. S. (2017). Drug delivery systems for prolonged duration local anesthesia. *Mater Today (Kidlington)* 20 22–31. 10.1016/j.mattod.2016.11.019 28970739 PMC5621744

[B72] Sauer R. S. Krug S. M. Hackel D. Staat C. Konasin N. Yang S. (2014). Safety, efficacy, and molecular mechanism of claudin-1-specific peptides to enhance blood-nerve-barrier permeability. *J. Control. Release* 185 88–98. 10.1016/j.jconrel.2014.04.029 24780266

[B73] Schmidt Y. Gaveriaux-Ruff C. Machelska H. (2013). mu-Opioid receptor antibody reveals tissue-dependent specific staining and increased neuronal mu-receptor immunoreactivity at the injured nerve trunk in mice. *PLoS One* 8:e79099. 10.1371/journal.pone.0079099 24278116 PMC3838372

[B74] Shankarappa S. A. Tsui J. H. Kim K. N. Reznor G. Dohlman J. C. Langer R. (2012). Prolonged nerve blockade delays the onset of neuropathic pain. *Proc. Natl. Acad. Sci. U.S.A.* 109 17555–17560. 10.1073/pnas.1214634109 23045676 PMC3491532

[B75] Staat C. Coisne C. Dabrowski S. Stamatovic S. M. Andjelkovic A. V. Wolburg H. (2015). Mode of action of claudin peptidomimetics in the transient opening of cellular tight junction barriers. *Biomaterials* 54 9–20. 10.1016/j.biomaterials.2015.03.007 25907035

[B76] Surana S. Tosolini A. P. Meyer I. F. G. Fellows A. D. Novoselov S. S. Schiavo G. (2018). The travel diaries of tetanus and botulinum neurotoxins. *Toxicon* 147 58–67. 10.1016/j.toxicon.2017.10.008 29031941

[B77] Tajdaran K. Shoichet M. S. Gordon T. Borschel G. H. (2015). A novel polymeric drug delivery system for localized and sustained release of tacrolimus (FK506). *Biotechnol. Bioeng.* 112 1948–1953. 10.1002/bit.25598 25850693

[B78] Thuenauer R. Muller S. K. Romer W. (2017). Pathways of protein and lipid receptor-mediated transcytosis in drug delivery. *Expert Opin. Drug Deliv.* 14 341–351. 10.1080/17425247.2016.1220364 27500785

[B79] Truong W. Cheng C. Xu Q. G. Li X. Q. Zochodne D. W. (2003). Mu opioid receptors and analgesia at the site of a peripheral nerve injury. *Ann. Neurol.* 53 366–375. 10.1002/ana.10465 12601704

[B80] Ubogu E. E. (2013). The molecular and biophysical characterization of the human blood-nerve barrier: current concepts. *J. Vasc. Res.* 50 289–303. 10.1159/000353293 23839247 PMC4030640

[B81] Vadhanan P. Tripaty D. K. Adinarayanan S. (2015). Physiological and pharmacologic aspects of peripheral nerve blocks. *J. Anaesthesiol. Clin. Pharmacol.* 31 384–393. 10.4103/0970-9185.161679 26330722 PMC4541190

[B82] Wang L. Hao Y. Li H. Zhao Y. Meng D. Li D. (2015). Co-delivery of doxorubicin and siRNA for glioma therapy by a brain targeting system: angiopep-2-modified poly(lactic-co-glycolic acid) nanoparticles. *J. Drug Target* 23 832–846. 10.3109/1061186X.2015.1025077 25856302

[B83] Wiley D. T. Webster P. Gale A. Davis M. E. (2013). Transcytosis and brain uptake of transferrin-containing nanoparticles by tuning avidity to transferrin receptor. *Proc. Natl. Acad. Sci. U.S.A.* 110 8662–8667. 10.1073/pnas.1307152110 23650374 PMC3666717

[B84] Wilson B. A. Ho M. (2014). Cargo-delivery platforms for targeted delivery of inhibitor cargos against botulism. *Curr. Top. Med. Chem.* 14 2081–2093. 10.2174/1568026614666141022094517 25335885 PMC4410985

[B85] Wolfe D. Mata M. Fink D. J. (2012). Targeted drug delivery to the peripheral nervous system using gene therapy. *Neurosci. Lett.* 527 85–89. 10.1016/j.neulet.2012.04.047 22565023 PMC3458184

[B86] Xia B. Lv Y. (2018). Dual-delivery of VEGF and NGF by emulsion electrospun nanofibrous scaffold for peripheral nerve regeneration. *Mater. Sci. Eng. C Mater. Biol. Appl.* 82 253–264. 10.1016/j.msec.2017.08.030 29025656

[B87] Xiao J. Zhai H. Yao Y. Wang C. Jiang W. Zhang C. (2014). Chrysin attenuates experimental autoimmune neuritis by suppressing immuno-inflammatory responses. *Neuroscience* 262 156–164. 10.1016/j.neuroscience.2014.01.004 24412705

[B88] Yan Y. Sun H. H. Hunter D. A. Mackinnon S. E. Johnson P. J. (2012). Efficacy of short-term FK506 administration on accelerating nerve regeneration. *Neurorehabil. Neural Repair* 26 570–580. 10.1177/1545968311431965 22291040

[B89] Yang S. Krug S. M. Heitmann J. Hu L. Reinhold A. K. Sauer S. (2016). Analgesic drug delivery via recombinant tissue plasminogen activator and microRNA-183-triggered opening of the blood-nerve barrier. *Biomaterials* 82 20–33. 10.1016/j.biomaterials.2015.11.053 26735170

[B90] Yosef N. Ubogu E. E. (2013). An immortalized human blood-nerve barrier endothelial cell line for in vitro permeability studies. *Cell Mol. Neurobiol.* 33 175–186. 10.1007/s10571-012-9882-7 23104242 PMC3568212

[B91] Zhang L. Zhou Y. Li G. Zhao Y. Gu X. Yang Y. (2014). Nanoparticle mediated controlled delivery of dual growth factors. *Sci. China Life Sci.* 57 256–262. 10.1007/s11427-014-4606-5 24430559

[B92] Zhang N. Chittasupho C. Duangrat C. Siahaan T. J. Berkland C. (2008). PLGA nanoparticle–peptide conjugate effectively targets intercellular cell-adhesion molecule-1. *Bioconjug. Chem.* 19 145–152. 10.1021/bc700227z 17997512 PMC2535939

[B93] Zhang W. Zhou G. Gao Y. Zhou Y. Liu J. Zhang L. (2017). A sequential delivery system employing the synergism of EPO and NGF promotes sciatic nerve repair. *Colloids Surf. B Biointerfaces* 159 327–336. 10.1016/j.colsurfb.2017.07.088 28806665

[B94] Zhang Y. Zhang W. Johnston A. H. Newman T. A. Pyykko I. Zou J. (2012). Targeted delivery of Tet1 peptide functionalized polymersomes to the rat cochlear nerve. *Int. J. Nanomed.* 7 1015–1022. 10.2147/IJN.S28185 22403485 PMC3292415

[B95] Zhao J. Zheng X. Fu C. Qu W. Wei G. Zhang W. (2014). FK506-loaded chitosan conduit promotes the regeneration of injured sciatic nerves in the rat through the upregulation of brain-derived neurotrophic factor and TrkB. *J. Neurol. Sci.* 344 20–26. 10.1016/j.jns.2014.06.005 24954089

[B96] Zwanziger D. Hackel D. Staat C. Bocker A. Brack A. Beyermann M. (2012). A peptidomimetic tight junction modulator to improve regional analgesia. *Mol. Pharm.* 9 1785–1794. 10.1021/mp3000937 22524793

